# Evidence for an evo‐devo‐derived hypothesis on three‐dimensional flower shape modularity in a tropical orchid clade

**DOI:** 10.1111/evo.14621

**Published:** 2022-10-01

**Authors:** Silvia Artuso, Alexander Gamisch, Yannick M. Staedler, Jürg Schönenberger, Hans Peter Comes

**Affiliations:** ^1^ Department of Environment and Biodiversity University of Salzburg Salzburg 5020 Austria; ^2^ Department of Botany and Biodiversity Research University of Vienna Vienna 1030 Austria

**Keywords:** 3D geometric morphometrics, *Bulbophyllum*, evo‐devo, flower shape modularity, high‐resolution X‐ray computed tomography, Orchidaceae, phylogenetic comparative methods

## Abstract

Covarying suites of phenotypic traits, or modules, are increasingly recognized to promote morphological evolution. However, information on how modularity influences flower diversity is rare and lacking for Orchidaceae. Here, we combine high‐resolution X‐ray computed tomography scanning with three‐dimensional geometric morphometrics and phylogenetic comparative methods to test various hypotheses about three‐dimensional patterns of flower evolutionary modularity in Malagasy *Bulbophyllum* orchids and examine rates and modes of module evolution. Based on the four evolutionary modules identified (i.e., sepals, lateral petals, labellum + column‐foot, and column‐part), our data support the hypothesis that both genetic‐developmental and functional adaptive factors shaped evolutionary flower trait covariation in these tropical orchids. In line with “evo‐devo” studies, we also find that the labellum evolved independently from the rest of the petal whorl. Finally, we show that modules evolved with different rates, and either in a neutral fashion (only column‐part) or under selective constraints, as likely imposed by pollinators. Overall, this study supports current views that modular units can enhance the range and rate of morphological evolution.

Morphological traits often do not evolve independently, but instead show different patterns of correlated evolution (e.g., Olson and Miller 1958; Armbruster and Schwaegerle 1996; Pigliucci and Preston 2004; Klingenberg and Marugán‐Lobón 2013; Adams and Felice 2014). Suites of traits that covary in an integrated manner but quasi‐independently from other suites of traits are called modules (Klingenberg 2008, 2010, 2014; Murren 2012; Armbruster 2014). Morphological modularity and integration are thus fundamental concepts of complex biological systems that can be studied at multiple levels (and units) of organization; however, the underlying processes may not necessarily operate at each level (Klingenberg 2008; Armbruster et al. 2014). For example, in microevolutionary studies (at the among‐individual and/or intraspecific levels), “variational” modules usually refer to traits that covary because they are coinherited and share the same genetic regulation, developmental pathway, and/or functional adaptation (Klingenberg 2008; Diggle 2014; Zelditch and Goswami 2021). By contrast, in macroevolutionary studies (e.g., across species or clades), “evolutionary” modularity is often more broadly defined, encompassing correlated trait evolution that may be driven not only by genetic‐developmental processes but also by correlational (e.g., directional and/or stabilizing) selection (Goswami 2006; Zelditch and Goswami 2021). Macroevolutionary studies of modularity across species or clades of animals have shown that modules can follow different evolutionary trajectories and may evolve at different rates (Goswami and Polly 2010; Felice and Goswami 2018; Bardua et al. 2020, 2019; see also Esteve‐Altava 2017 for a review). As a consequence, the important role of modular organization in fostering morphological evolution and species diversification in animals is increasingly recognized (Bissell and Diggle 2010; Felice et al. 2018; Bardua et al. 2020, 2019). By contrast, little is known about the influence of modularity on the evolution of floral morphological diversity in plants, and whether modular integration either facilitates or constraints floral morphological disparity (Armbruster 2014; Armbruster et al. 2014; Dellinger et al. 2019; Reich et al. 2020). Concomitantly, few studies to date have examined floral patterns of evolutionary modularity and integration across angiosperm taxa and macroevolutionary timescales (but see Dellinger et al. 2019; Reich et al. 2020).

Flowers are ideal objects for the study of evolutionary modularity, as they are formed by different organ classes that often interact in multiple ways with pollinators (Bowman et al. 2012; Armbruster 2014; Armbruster et al. 2014; Diggle 2014; Irish 2017). According to Diggle (2014), three main hypotheses of flower modularity can be distinguished: (1) the “efficiency” hypothesis assumes that traits involved in pollen removal and deposition, whether directly (e.g., anthers [androecium], carpels [gynoecium]) or indirectly (e.g., corolla tube), form one or several efficiency modules that are distinct from those related to pollinator attraction (Rosas‐Guerrero et al. 2011); (2) the “attraction” hypothesis proposes that flowers are divided between attracting structures and strictly reproductive ones (e.g., sepals and/or petals vs. anthers and carpels) (Esteve‐Altava 2017); and (3) the “developmental” hypothesis posits that flower parts sharing the same regulatory (developmental and/or genetic) pathway form an independent module (Reich et al. 2020). Assuming a classical “whorl‐based” flower structure, this hypothesis generally divides the flower in four modules of sepals (outer perianth whorl), petals (inner perianth whorl), anthers, and carpels (Diggle 2002; Reich et al. 2020). To date, the efficiency hypothesis has received the most support in studies of variational modularity at the intraspecific level (e.g., *Mimulus luteus* var. *luteus*: Carvallo and Medel [2005]; Solanaceae spp.: Pérez et al. [2007]; *Nicotiana* spp.: Bissell and Diggle [2008, 2010]; *Ipomoea* spp.: Rosas‐Guerrero et al. [2011]; *Ruellia humilis* (Acanthaceae): Heywood et al. [2017]; *Stylidium* spp.: Armbruster and Wege [2019]), but also in studies of evolutionary modularity across species (Melastomataceae: Dellinger et al. [2019]; Ericaceae: Reich et al. [2020]); however, some intraspecific studies also found support for the attraction hypothesis (e.g., Tucić et al., 2013) or the developmental hypothesis (e.g., Herrera et al. 2002; Armbruster and Wege 2019), whereas the latter also received support in the macroevolutionary study of Reich et al. (2020) with regard to pollination generalist species. That said, no study to date has tested the extent to which developmental‐genetic factors or functional adaptations shape flower modularity and integration in members of Orchidaceae, neither within species (variational modularity) or at the interspecific level (evolutionary modularity).

With about 26,000 species (WCSP 2021), orchids are one of the most species‐rich families of angiosperms and particularly well‐known for a close relationship between flowers and pollinators (e.g., Johnson et al. 1998; Cozzolino and Widmer 2005; Sletvold et al. 2010). There are at least three distinctive flower characteristics that set orchids apart from most other plant taxa: (1) the pollen usually stays in masses called pollinia; (2) the anther(s) and carpels are fused into common structure called column or gynostemium; and (3) the median tepal (petal) of the inner perianth whorl often forms a labellum or lip, which is positionally abaxial in resupinated flowers, and which often acts as a visual attractant and/or landing stage for pollinating insects (Rudall and Bateman 2002; Gravendeel and Dirks‐Mulder 2015; Endress 2016). Moreover, column and labellum (together conferring zygomorphy) are commonly invoked as a “vital pairing” for interacting with pollinators (cf. Rudall and Bateman 2002). A joint functional role of labellum and column is particularly evident in *Bulbophyllum* Thouars (Epidendroideae), featuring a well‐known “see‐saw” pollination mechanism (e.g., Ridley 1890; Teixeira et al. 2004; Tan 2009; Nunes et al. 2014; Ong and Tan 2011). Thus, in typically fly (or rarely bee, wasp) pollinated species of this pantropical genus (about 2400 spp.; Sieder et al. 2009), the pollinator usually moves along the hinged and motile labellum and is forced against the column for pollinia deposition or removal (e.g., Chen and Gao 2011; Chen et al. 2014; Ong and Tam 2019; Hu et al. 2020; see Fig. 1 for details). Hence, at least for this genus, the efficiency hypothesis of modularity would predict that labellum and column together form a distinct unit that evolved separately from both sepals and the remaining petals, which are likely to act primarily as visual attractants (Spaethe et al. 2007; Rakosy et al. 2012; Kowalkowska et al. 2015, 2017; Dodson 2020; but see Hu et al. 2020).

**Figure 1:**
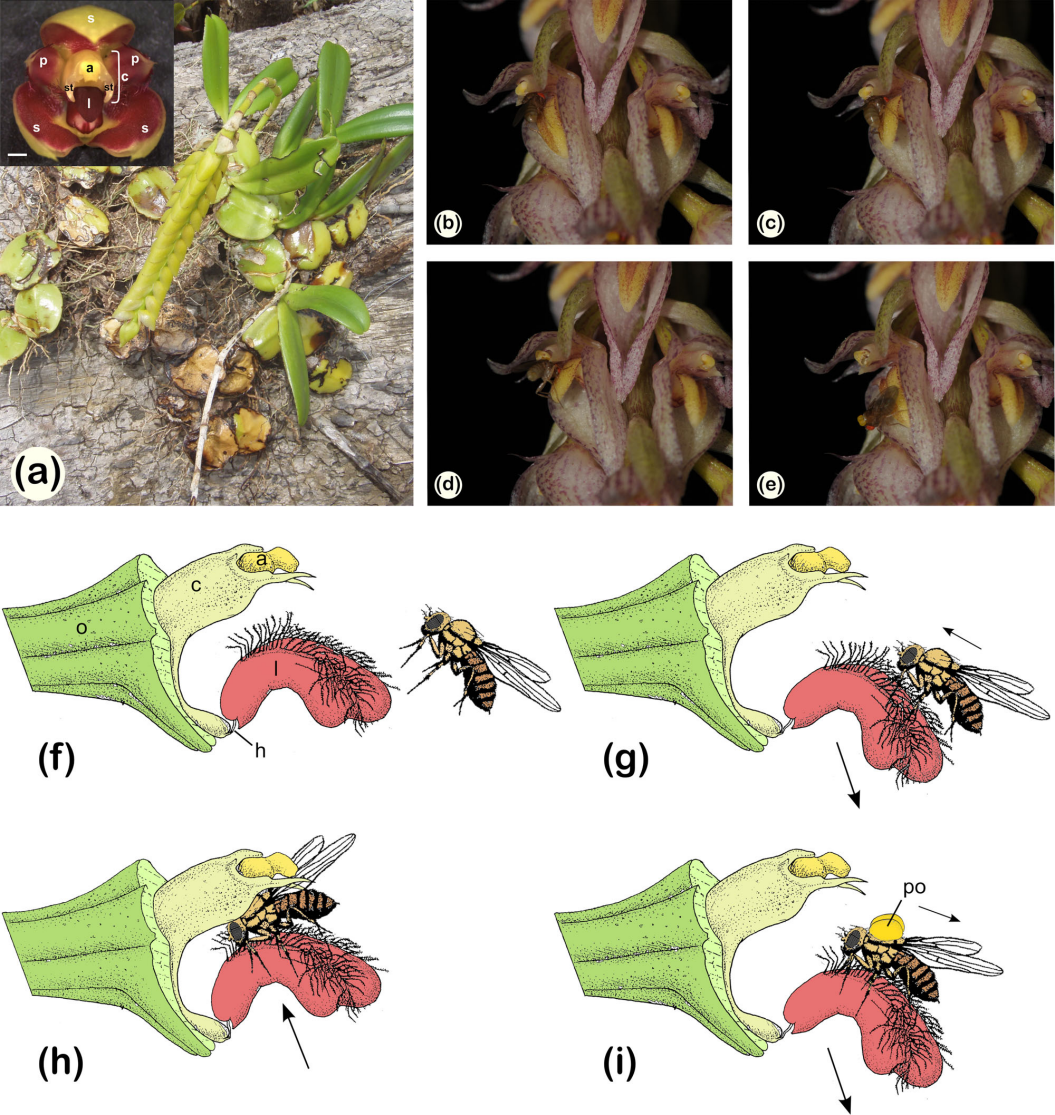
**Figure 1**. Habitus, flower morphology, and pollination of *Bulbophyllum*. (a) Habitus and flower detail of Malagasy *Bulbophyllum cirrhoglossum* (Clade *A*). Photographs by Anton Sieder (habitus) and Alexander Gamisch (close‐up). (b–e) Sequence showing a *Drosophila* fly removing pollinia from *Bulbophyllum lilacinum*. Photographs by Poh T. Ong. (f–i) Illustration of the “see‐saw” pollination mechanism of *Bulbophyllum* flowers (following Chen and Gao 2011), with arrows indicating the direction of movement of the labellum during pollinia removal. (f) A flower with sepals and petals removed. The movable labellum is hinged at the base of the column. (g) The pollinator (here a fly) lands on the labellum, which moves down under the weight of the insect. (h) The fly crawls toward the base of the labellum; once the balance point is passed, the labellum moves up to its original position, forcing the fly against the column; at this point, the pollinia are removed by sticking to the insect's thorax dorsally. (i) The pollinator leaves the flower and takes the pollinia away. a, anther cap; c, column (gynostemium); h, hinge (ligament); l, labellum (lip); o, ovary; p, petal; po, pollinia; s, sepal; st, stelidium. The sketches (f–i) were prepared by Alexander Gamisch and Silvia Artuso, based on a drawing of Malagasy *B. quadrifarium* by Juliet Beentje (Kew Gardens, U.K.) and a fly sketch (J*Fly 2021), and following the schematic drawings of Chen and Gao (2011) for a bee‐pollinated Asian species (*B. ambrosia*). Scale bars, 1 mm.

Notably, there is also increasing evidence from molecular evolutionary developmental (“evo‐devo”) studies to suggest a tripartite (rather than bipartite sepal vs. petal) modularization of the orchid perianth, comprising sepals, lateral petals, and labellum, as specified by the differential expression of floral homoeotic (MADS‐box) genes (e.g., Mondragón‐Palomino and Theißen 2008, 2009, 2011; Bateman and Rudall 2019; Wang et al. 2019). Therefore, it has been argued that these different kinds of perianth organs could evolve “individually and thus often in dramatically different ways in response to selection by pollinators or by genetic drift” (cf. Mondragón‐Palomino and Theißen 2009). However, this “evo‐devo” hypothesis of orchid flower modularity has yet to be tested across species and clades. Also, to our knowledge, no previous study has tested hypotheses of flower modularization by taking explicitly into account evidence from developmental genetics.

In this study, we focus on a phylogenetically well‐defined lineage of *Bulbophyllum* from Madagascar, termed “Clade *A*” (about 50 spp.; crown age about 11.5 million years ago [Ma]; Gamisch et al. 2021). In general, these species (like Malagasy *Bulbophyllum* as a whole; about 210 spp.; Sieder et al. 2009) display one or few many flowered inflorescences with small (about 0.5–2 cm) and usually resupinated flowers (except *Bulbophyllum alexandrae*) that vary in shape, size, and color (Cribb and Hermans 2009). Even if few, field observations indicate that *Bulbophyllum* species from Madagascar are mostly pollinated by small Dipteran flies (Humeau et al. 2011; Pailler and Baider 2020; Hermans et al. 2021). These likely interact with the flower in the same specialized way as typical for other fly‐pollinated species of this genus elsewhere (see above; Fig. 1).

In a recent study, Artuso et al. (2021) combined high‐resolution X‐ray computed tomography (HRX‐CT) scanning (Staedler et al. 2018) with landmark (LM)‐based three‐dimensional (3D) geometric morphometrics (GM; Bookstein 1997; Klingenberg 2010) (see Fig. 2) and multivariate phylogenetic comparative methods (PCMs; Garamszegi 2014) to study whole‐flower shape evolution across 38 species of Clade *A*, using a time‐calibrated phylogeny (Gamisch et al. 2021). Based on this previous 3D flower scan/LM dataset, the major aim of the present work is to evaluate a priori hypotheses of evolutionary flower modularity and integration as well as the modes and rates of module diversification. Specifically, we adopted a maximum likelihood (ML) approach (Goswami and Finarelli 2016) to primarily test whether evolutionary patterns of modularization in these tropical orchids conform with one of the commonly invoked “attraction,” “efficiency,” or “developmental” (“whorl‐based”) hypotheses of flower modularity (Diggle 2014), or the specific “developmental‐genetic” (“evo‐devo”) model of the orchid flower (e.g., Mondragón‐Palomino and Theißen 2009). In addition, based on the best‐fit hypothesis, we employed a penalized likelihood (PL) approach (Clavel et al. 2019) to fit PCM‐based models of trait evolution to each module identified across the Clade *A* phylogeny. Finally, we compared rates of evolution and levels of morphological variation (disparity) among those modules to determine the extent to which they may have evolved following different modes and/or rates, leading to differences in disparity. Overall, these analyses provide novel insights into the evolutionary patterns of modularity and integration in orchids and clarify the extent to which different subtraits independently responded to different evolutionary processes in Clade *A*.

**Figure 2:**
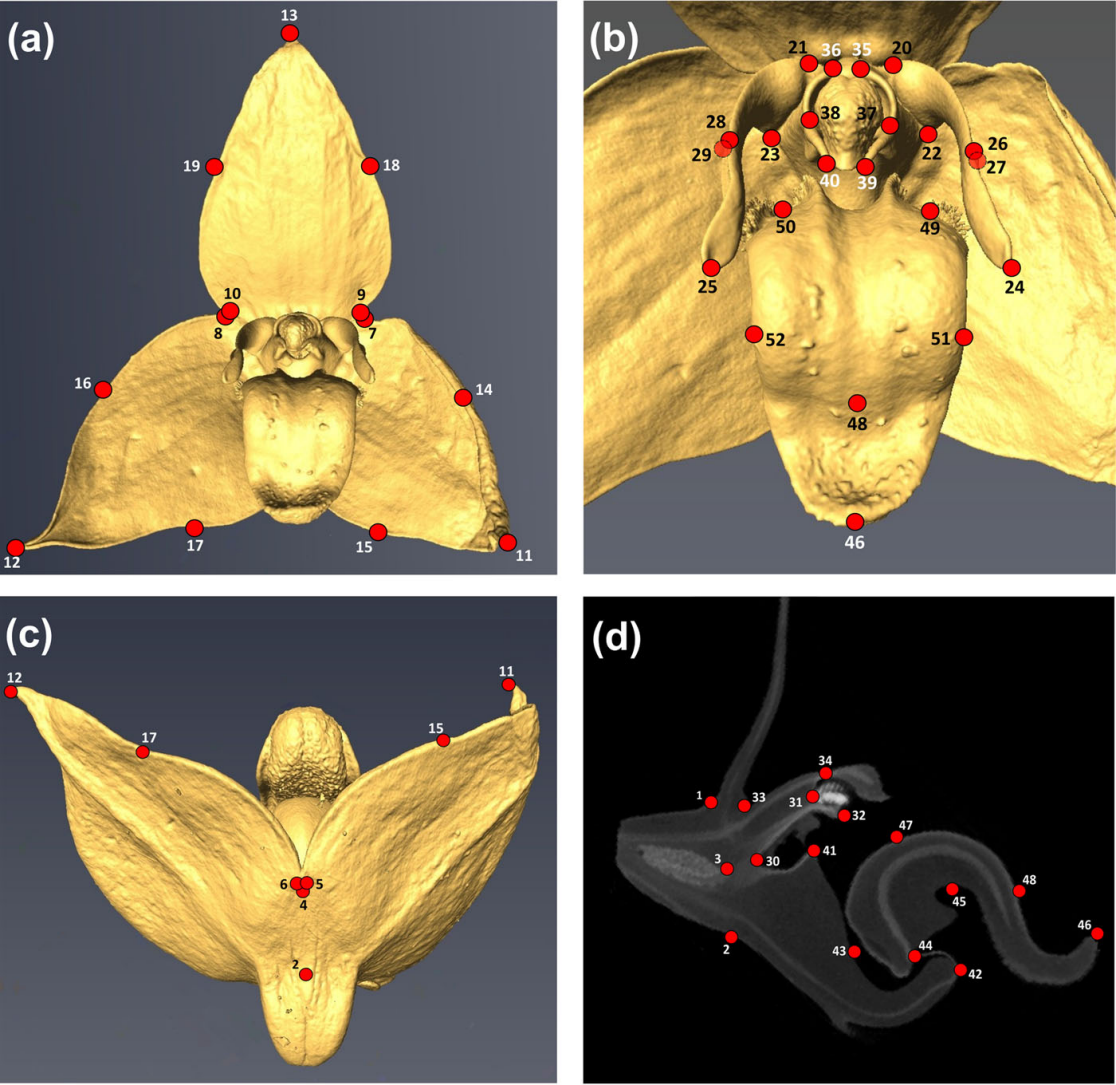
**Figure 2**. Landmarks (LMs 1–52; red dots) placed onto high‐resolution X‐ray computed tomography (HRX‐CT) scans of an exemplary flower of Malagasy *Bulbophyllum* (*B. francoisii* of sect. *Elasmotopus*) (see also Artuso et al. 2021). (a) Front view of resupinate flower at anthesis, in more or less horizontal orientation. (b) Close‐up of frontal view. (c) Flower seen from below (abaxial side). (d) Median (longitudinal) section. Refer to Table S2 for description of LMs and their assignment to different modules per evolutionary modularity hypothesis.

## Materials and Methods

### PLANT MATERIAL AND CLADE A PHYLOGENY

We used a 3D flower shape dataset (previously described in Artuso et al. 2021) for 38 (out of 50) Clade *A* species, representing four multi‐species sections (*Alcistachys*, *Bifalcula*, *Calamaria*, and *Kainochilus*) and the monotypic sect. *Polyradices* (see Table S1). Our Clade *A* phylogeny (Fig. S1) was obtained from a time‐calibrated maximum clade credibility (MCC) tree of Malagasy *Bulbophyllum* (179/210 spp.), based on three nuclear and five plastid gene regions (Gamisch et al. 2021). The MCC chronogram was pruned to the 38 species considered here, using the R package phytools version 0.6.99 (Revell 2012). See Artuso et al. (2021) for further details on flower material and phylogenetic relationships among sections and divergence times in Clade *A*.

### HRX‐CT SCANNING, 3D FLOWER MODELING, AND LANDMARKING

Detailed information about the procedures followed for HRX‐CT scanning and 3D‐GM analyses can be found in Artuso et al. (2021). In brief, for each of the 38 species, one flower was collected, using either ethanol‐preserved material (36 spp.) or herbarium specimens (*Bulbophyllum maculatum*, *Bulbophyllum rubrum*); the latter were rehydrated following Erbar (1995). The HRX‐CT scanning followed Staedler et al. (2013), using xmreconstructor version 8.1.6599 (XRadia Inc.) for the reconstruction of 3D image stacks from the raw data. For the 3D shape analysis, 52 LMs were placed onto each 3D‐flower model, using amira version 6.0 (Template Graphics Software Inc., San Diego, CA; see Fig. 2 and Table S2). Accuracy of LM placement and intraobserver error assessment, along with a pilot study of intra‐ vs. interspecific shape variation in the sister species *Bulbophyllum bicoloratum*/*Bulbophyllum occultum*, can be found in the Supporting Information of Artuso et al. (2021).

### MODULARITY HYPOTHESIS FRAMEWORK

**Figure 3:**
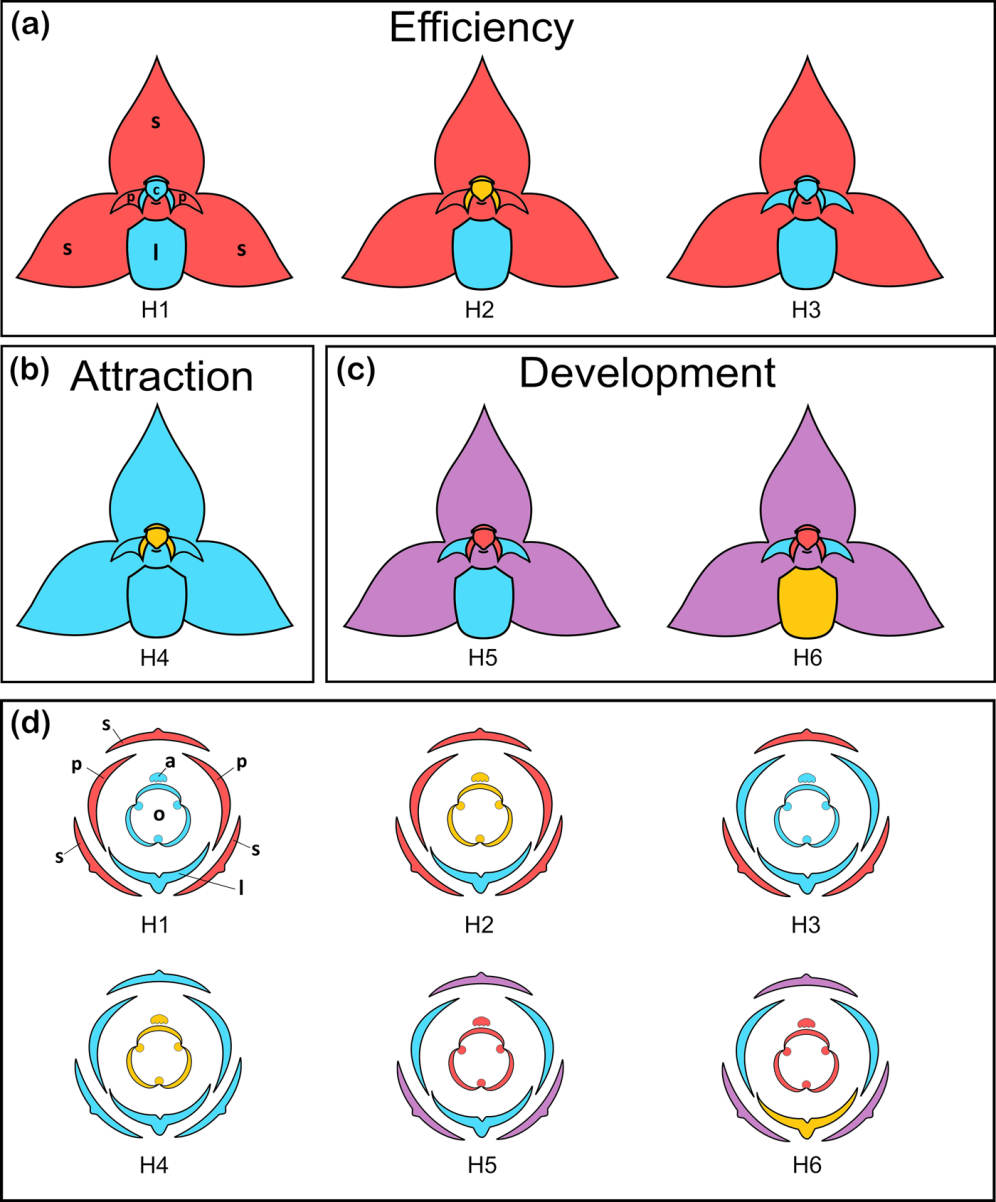
**Figure 3**. Illustration of the six major evolutionary modularity hypotheses (H1–6) tested for flowers of 38 species of Malagasy *Bulbophyllum* Clade *A*, as divided into three categories: (a) efficiency, (b) attraction, and (c) development (see text and Tables 2 and S3 for further explanation). (a–c) Front view of resupinate flowers at anthesis, in horizontal orientation. Flower organs are colored according to the module they belong to. (d) Corresponding flower diagrams for each of the six modularity hypotheses. Note, there are four additional (sub‐)hypotheses that assign the labellum with the column‐foot, that is, H2*, H4*, H5*, and H6* (the latter is illustrated in Fig. S2). Abbreviations of flower organs: a, anther; c, column (gynostemium); l, labellum (lip); o, ovary; p, petal; s, sepal

As illustrated in Figure 3, we tested six major flower evolutionary modularity hypotheses (H1–6), divided into three categories: “efficiency” (H1–3), “attraction” (H4), and “developmental” (H5, H6), respectively (see Table S2 for details on the assignment of LMs to the different modules under each hypothesis). For the “efficiency” category (Fig. 3a), hypotheses H1–3 reflected different modular divisions with emphasis on floral organs directly involved in pollinia deposition and/or removal, taking into consideration that these flowers are likely visited by the same functional group of pollinators (small Dipteran flies). In detail, considering the crucial role of the movable labellum in forcing the pollinator against the column (see in the introductory text; Fig. 1), H1 treated labellum + column (and its appendages/stelidia) together as part of an “efficiency” module versus sepals + lateral petals; by contrast, H2 regarded labellum and column as two separate “efficiency” modules against the remainder; finally, as the lateral petals might also guide pollinators along the main floral axis to the column (Nunes et al. 2015; but see Pakum et al. 2019), H3 grouped lateral petals + labellum + column into a single “efficiency” module versus the sepals. For the “attraction” category (Fig. 3b), we designed a single two‐module hypothesis (H4), separating the strictly reproductive part (column) from a supposedly attractive remainder (sepals + lateral petals + labellum). Finally, for the “developmental” category (Fig. 3c), we first specified a three‐module, “developmental‐morphological” or “whorl‐based” hypothesis (H5), reflecting the classic three‐whorl structure of the orchid flower, that is, sepals versus petals (including labellum) versus column (e.g., Arditti and Harrison 1979; Rudall and Bateman 2002). Alternatively, we newly defined a four‐module, “developmental‐genetic” or “evo‐devo” hypothesis (H6), taking evidence for an independent genetic control of the labellum into account, that is, sepals versus lateral petals versus labellum versus column (e.g., Mondragón‐Palomino and Theißen 2011).

In addition, we investigated further refinements of a subset of hypotheses by considering that the movable labellum of *Bulbophyllum* is typically joined to the tip of the column‐foot through a ligament (Vermeulen, 2008). As a result, the labellum can move from an open to a closed position, whereby its base closely adjoins the column (Teixeira et al. 2004; Liu et al. 2010; Kowalkowska et al. 2017). We therefore hypothesized that the shape of the column‐foot could correlate more with that of the labellum than with the rest of the column or “column‐part” (sensu Rudall and Bateman 2002). Accordingly, for each of the four major hypotheses that treated labellum and column separately (H2, H4–6), we tested a “counterpart” hypothesis (H2*, H4* etc.) by assigning two LMs of the column‐foot to those of the labellum (LM 42: attachment point of the ligament to the column; LM 43: internal rim of the column‐foot; see Fig. 2 and Table S2), resulting in a total of 10 hypotheses. Overall, however, it needs to be emphasized that especially the “efficiency” and “attraction” hypotheses are not always mutually exclusive, as a given floral organ might be considered to represent either kind of module (e.g., the lateral petals and labellum might “efficiently” interact with pollinators while also serving as visual attractants; see above).

### STATISTICAL ANALYSES

#### 3D‐GM dataset analyses

The following preparatory GM analyses were performed on the high‐dimensional LM dataset using geomorph version 3.3.1 (Adams et al. 2020) in an R environment (version 4.0.3; R Core Team 2021), namely (1) a Generalized Procrustes Analysis (GPA) superimposition (to separate shape from size, position and orientation); (2) the removal of potential effects of asymmetry from the data (function *bilat.symmetry*); and (3) an evaluation of potential evolutionary allometric effects of size on the shape data (function *procD.pgls*), that is, taking phylogenetic relationships into account. Because we found no significant effect of size on shape (*P* = 0.51), we did not apply any allometric correction to the data.

#### Evolutionary modularity hypothesis testing

Prior to hypothesis testing, we first assigned each LM to the different modules per hypothesis (see Table S2), thereby using the symmetric component of the GPA‐derived coordinates to conserve information about the connection of the different subsets (“partitions”) and their relative sizes (Klingenberg 2009). Based on the resulting 3D dataset, we adopted three approaches to compare the 10 evolutionary modularity hypotheses outlined above. First, we quantified the degree of modularity in geomorph (function *modularity.test*), using the covariance ratio (CR) coefficient, which has proven to be less sensitive to small sample sizes with changing variables (Adams 2016). The CR value (ranging from 0 to infinity) quantifies the degree of modularity (namely, independence between modules) relative to a random assignment of the LMs to each partition (null hypothesis); an observed CR value significantly lower compared to the random distribution (based on 999 permutations) is considered strong support for a given modularity hypothesis (Adams 2016). Second, for those hypotheses receiving significant support by the CR test, we also used the geomorph function *compare.cr* to calculate “effect size” measures (*Z*_CR_), with more negative values representing a stronger modular signal (Adams and Collyer 2019). Finally, we generated a correlation matrix of the individual Procrustes‐fitted LM coordinates in paleomorph version 0.1.4 (function *dotcorr*; Lucas and Goswami 2017) for a subsequent analysis of modularity in the R package emmli version 0.0.3 (Goswami and Finarelli 2016). This program allows for the inclusion of high‐dimensional data and uses an ML approach for the direct comparison of different modularity hypotheses by taking different model parametrizations into account. Thus, for each modularity hypothesis, emmli explores whether correlation (“integration”) coefficients (*ρ*s) are either the same or different (“separate”) within and/or between modules (Goswami and Finarelli 2016). For our dataset, this resulted in a total of 32 parameterization schemes specified across the 10 hypotheses (two or four per hypothesis; labeled “a–d”), plus testing a null model of “no modularity.” The best‐fit hypothesis was determined through the lowest corrected Akaike information criterion (AICc) score (Goswami and Finarelli 2016). For comparison, we also repeated all the above analyses (geomorph: CR, *Z*_CR_; paleomorph/emmli) by taking phylogenetic relationships among our 38 study species into account. For the geomorph analyses, we used the function *phylo.modularity*, which assumes by default a Brownian motion (BM) model of trait evolution, to generate phylogenetically corrected covariance matrices; for the emmli analysis, we computed a likewise‐corrected correlation matrix in R, using phylogenetic independent contrasts (Felsenstein 1985).

#### Quantifying degrees of within‐ and among‐module integration

We used the ML correlation (*ρ* values, as estimated by emmli under the best‐fit modularity hypothesis) to measure degrees of integration within and between the flower organ evolutionary modules identified (Goswami and Finarelli 2016). Results are reported for both uncorrected and phylogenetically corrected datasets.

#### Testing modes and rates of module evolution

To test whether modes of evolution differ among modules, we used the PL framework in rpanda version 1.6 (Morlon et al. 2016; function *fit_t_pl*) to fit three commonly used PCM models of trait evolution (i.e., BM; single‐optimum Ornstein–Uhlenbeck [OU]; early burst [EB]) directly on the 3D Procrustes‐fitted LM coordinates of each of the four evolutionary modules identified under H6*. In brief, the BM model describes a constant‐rate “random walk” process, and is often considered the de facto null hypothesis of neutral continuous trait evolution (e.g., Felsenstein 1985; Felice et al. 2018; Blomberg et al. 2020; Soul and Wright 2021; see also Artuso et al. 2021); the OU model constrains the BM process by including an evolutionary force (“pull”) toward an optimal trait value (Hansen 1997; Beaulieu et al. 2012; Cressler et al. 2015); and the EB model describes an initially rapid phenotypic evolution followed by a slowdown (Harmon et al. 2010; see also Artuso et al. 2021 for more details). Following Clavel et al. (2019), we used the Generalized Information Criterion (GIC) (function *GIC*) based on randomized 100 trees of the pruned (38 spp.) chronogram of Clade *A* (Fig. S1) to further evaluate the model fit for each module. For those modules following an OU process, we calculated the “rate of adaptation” parameter, *α*, and its associated “phylogenetic half‐life” (*t*_1/2_), as given by the formula (Hansen 1997, 2014): *t*_1/2_ = ln2/*α*, assuming a tree height of about 11.5 million years (Myr), based on the crown age of Clade *A* (Gamisch et al. 2021; see also Fig. S1). Phylogenetic half‐lives are usually interpreted as the average time it takes a species to evolve halfway from its ancestral state (here: module shape) toward the optimum (see also Artuso et al. 2021), thereby indicating the strength of the OU process acting on the species. When *α* is zero and/or *t*_1/2_ is larger relative to tree height, the OU model collapses to the BM model (Beaulieu et al. 2012; Cooper et al. 2016).

We compared the multivariate net rates of shape evolution (*σ^2^*_mult_) among the four evolutionary modules using the geomorph function *compare.multi.evol.rates* (Denton and Adams 2015). Significant differences in the rates were tested by simulating the 38‐tip data of the Clade *A* chronogram, assuming (by default) a BM process (Denton and Adams 2015).

#### Levels of disparity

Levels of morphological disparity among the four evolutionary modules were investigated using the geomorph function *morphol.disparity*. For each module, morphological disparity was standardized by dividing the Procrustes variance by the number of LMs. Significant differences in disparity were evaluated by comparing the means of the modules’ variances, using Tukey's HSD (honestly significant difference) post hoc test in R version 4.0.3, with *P* values adjusted for multiple comparisons (see Bardua et al. 2019).

## Results

### EVOLUTIONARY MODULARITY HYPOTHESIS TESTING

Based on the CR test in geomorph for phylogenetically uncorrected covariance matrices (Table 1), all 10 evolutionary modularity hypotheses (for details see *Materials and Methods* and Fig. 3) were statistically highly significant (all *P* values = 0.001) but nonetheless differed in their strength in terms of CR values (range: 0.595–0.757). The four‐module developmental‐genetic (“evo‐devo”) hypothesis H6 (sepals, lateral petals, labellum, column), as well as its counterpart, H6*, treating column‐foot and labellum together, received the lowest CR values (both 0.595), whereas the efficiency hypothesis H1 (labellum + column vs. sepals + lateral petals) performed worst (CR = 0.757). In contrast, by taking phylogenetic species relationships into account (Table 1), only four of the 10 hypotheses received statistical support at the 5% level; nonetheless, H6 and especially H6* performed best (CR = 0.815 and 0.786, respectively; both *P* values < 0.001). In terms of effect size measures (*Z*_CR_), hypotheses H6 and H6* also performed best by receiving the most negative values (Table 1). However, for the uncorrected dataset, H6 performed better than H6* (*Z*_CR_ = –7.63 vs. –7.18), whereas the reverse was true if phylogeny was taken into account (*Z*_CR_ = –4.47 vs. –4.93). By contrast, in line with the CR test, the ML‐based modeling approach in emmli for uncorrected (Table 2) and phylogenetically corrected data (Table S3) consistently provided by far the best fit for H6* upon proposing separate correlation coefficients (*ρ*s) within and between the four modules of this hypothesis (AICc = –2235.35 and –2404.74, respectively; each with model posterior probability, PP = 1.0; see parameterization scheme H6*–d in Tables 2 and S3). Hence, when taken together, the developmental‐genetic (“evo‐devo”) (sub‐)hypothesis H6*, with joint treatment of labellum + column‐foot (see Fig. S2), was clearly the preferred one, as supported by the CR test, the phylogenetically corrected *Z*_CR_ measures, and especially the emmli results.

**Table 1:** Flower evolutionary modularity tests for 38 species of *Bulbophyllum* Clade *A*, using the covariance ratio (CR) coefficient and the effect size measure (*Z*_CR_), as implemented in geomorph

		Uncorrected			Phylogenetically Corrected		
No.^1^	Modularity Hypothesis	CR	*P*	*Z*_CR_	CR	*P*	*Z*_CR_
1	Efficiency	0.757	0.001	–5.35	0.938	0.063	NA
2	Efficiency	0.666	0.001	–5.23	0.981	0.116	NA
2*	Efficiency	0.665	0.001	–5.66	0.934	0.029	–2.00
3	Efficiency	0.734	0.001	–5.14	1.021	0.312	NA
4	Attraction	0.751	0.001	–4.39	0.971	0.156	NA
4*	Attraction	0.700	0.001	–4.38	0.922	0.054	NA
5	Development	0.701	0.001	–5.99	0.970	0.075	NA
5*	Development	0.682	0.001	–5.97	0.937	0.027	–2.06
**6**	Development	**0.595**	**0.001**	**–7.63**	**0.815**	**0.001**	**–4.47**
6*	Development	**0.595**	**0.001**	**–7.18**	**0.786**	**0.001**	**–4.93**

*Note*: Results are presented for uncorrected and phylogenetically corrected covariance matrices, respectively. For the CR tests, the two lowest (and significant) values are highlighted in bold, and for the *Z*_CR_ tests, the two most negative values. Note that the *Z*_CR_ test was only performed for hypotheses with significant CR values (*P* < 0.05). See text, Tables 2 and S3 as well as Figures 3 and S2 for further descriptions and illustrations of the evolutionary modularity hypotheses.

NA = not analyzed.

^1^Numbers with an asterisk indicate those modularity hypotheses that assign landmarks (LMs) of the column‐foot to the labellum.

**Table 2:** emmli‐derived results for a total of 10 evolutionary flower modularity hypotheses, plus a null model of “no modularity”, tested for the phylogenetically uncorrected three‐dimensional landmark dataset of *Bulbophyllum* Clade *A* (38 spp.)

Hyp. No.^1^	Category	Parameter Scheme^2^	Modular Structure Description	No. of Modules	*MaxL*	*K*	AICc	ΔAICc	Model PP
0	No modularity	–	–	–	778.313	2	–1552.617	682.736	5.57 × 10^–149^
1	Efficiency		M1 [sepals + lateral petals], M2 [labellum + column]	2					
		a			823.543	3	–1641.067	594.287	8.96 × 10^–130^
		b			839.172	4	–1670.314	565.039	2.01 × 10^–123^
2	Efficiency		M1 [sepals + lateral petals], M2 [labellum], M3 [column]	3					
		a			875.282	3	–1744.545	490.808	2.64 × 10^–107^
		b			986.705	5	–1963.365	271.988	8.68 × 10^–60^
		c			882.324	5	–1754.603	480.750	4.04 × 10^–105^
		d			993.748	7	–1973.411	261.942	1.32 × 10^–57^
2*	Efficiency		M1 [sepals + lateral petals], M2 [labellum (+ column‐foot)], M3 [column‐part]	3					
		a			892.601	3	–1779.184	456.169	8.79 × 10^–100^
		b			1046.760	5	–2083.475	151.879	1.05 × 10^–33^
		c			910.294	5	–1810.543	424.811	5.67 × 10^–93^
		d			1064.453	7	–2114.821	120.532	6.71 × 10^–27^
3	Efficiency		M1 [sepals], M2 [lateral petals + labellum + column]	2					
		a			802.668	3	–1599.318	636.035	7.7 × 10^–139^
		b			806.125	4	–1604.219	631.135	8.93 × 10^–138^
4	Attraction		M1 [sepals + lateral petals + labellum], M2 [column]	2					
		a			846.802	3	–1687.585	547.769	1.13 × 10^–119^
		b			935.815	4	–1863.601	371.753	1.88 × 10^–81^
4*	Attraction		M1 [sepals + lateral petals + labellum (+ column‐foot)], M2 [column‐part]	2					
		a			870.104	3	–1734.190	501.163	1.49 × 10^–109^
		b			1012.464	4	–2016.898	218.456	3.66 × 10^–48^
5	Development		M1 [sepals], M2 [petals, including labellum], M3 [column]	3					
		a			887.608	3	–1769.197	466.156	5.96 × 10^–102^
		b			943.916	5	–1877.787	357.567	2.27 × 10^–78^
		c			895.484	5	–1780.923	454.431	2.1 × 10^–99^
		d			951.793	7	–1889.500	345.853	7.92 × 10^–76^
5*	Development		M1[sepals], M2 [petals, incl. labellum (+ column‐foot)], M3 [column‐part]	3					
		a			897.199	3	–1788.380	446.974	8.73 × 10^–98^
		b			1006.249	5	–2002.453	232.900	2.67 × 10^–51^
		c			915.273	5	–1820.501	414.853	8.24 × 10^–91^
		d			1024.323	7	–2034.562	200.792	2.50 × 10^–44^
6	Development		M1 [sepals], M2 [lateral petals], M3 [labellum] M4 [column]	4					
		a			972.915	3	–1939.812	295.541	6.67 × 10^–65^
		b			1024.935	6	–2037.806	197.548	1.27 × 10^–43^
		c			1000.828	8	–1985.546	249.807	5.69 × 10^–55^
		d			1052.847	11	–2083.494	151.860	1.06 × 10^–33^
6*	**Development**		**M1 [sepals], M2 [lateral petals], M3 [labellum (+ column‐foot)], M4 [column‐part]**	**4**					
		a			1002.962	3	–1999.907	235.447	7.47 × 10^–52^
		b			1085.467	6	–2158.870	76.484	2.47 × 10^–17^
		c			1046.273	8	–2076.437	158.917	3.10 × 10^–35^
		**d**			**1128.777**	**11**	**–2235.353**	**0**	**1.000**

*Note*: Each modularity hypothesis was tested for alternative parameterizations (a, b; or a–d), proposing that correlation (“integration”) coefficients (*ρ* values) are the same or different within and/or between modules (Goswami and Finarelli 2016). The best‐fit hypothesis, as determined through the lowest corrected Akaike Information Criterion (AICc) score, is highlighted in bold, that is, the developmental (“evo‐devo”) hypothesis H6*, with labellum and column‐foot treated together, and hypothesizing different *ρ* values within and between modules (H6*–d). For each hypothesis, module numbers (M1, M2, etc.) reflect the partitioning of landmarks, as detailed in Table S2. Corresponding results based on the phylogenetically corrected dataset can be found in Table S3.

*MaxL* = maximum log‐likelihood; *K* = model parameters; AICc = finite sample corrected Akaike Information Criterion; ΔAICc = difference in AICc value between the best model and the model being compared; Model PP = model posterior probability (note, same as model log‐likelihood; not shown).

^1^Hyp. No., modularity hypothesis number; those with an asterisk indicate hypotheses that assign landmarks (LMs) of the column‐foot to the labellum.

^2^Parameterization scheme of correlation (“integration”) coefficients (*ρ* values) within and between modules (with emmli connotation in italics): a, same within‐module *ρ*s and same between‐module *ρ*s (*same.Mod + same.between*); b, separate within‐module *ρ*s and same between‐module *ρ*s (*sep.Mod + same.between*); c, same within‐module *ρ*s and separate between‐module *ρ*s (*same.Mod + sep.between*); d, separate within‐module *ρ*s and separate between‐module *ρ*s (*sep.Mod + sep.between*).

### DEGREES OF WITHIN‐ AND BETWEEN‐MODULE INTEGRATION

Table 3 summarizes emmli‐derived ML estimates of within‐ and between‐module correlations (*ρ* values) for uncorrected and phylogenetically corrected datasets, respectively. Accordingly, the column‐part evolutionary module (i.e., column without foot) represented the most strongly integrated organ structure (range of *ρ* values across uncorrected and corrected data: 0.46–0.53), followed by the labellum + column‐foot (0.43), the lateral petals (0.32–0.36), and the sepals (0.28). By comparison, and not unexpectedly, intermodule correlations were much lower (range of *ρ* values for all between‐module correlations: 0.12–0.27; Table 3).

**Table 3:** Maximum likelihood (ML) estimates of correlation (“integration”) coefficients (*ρ* values) within and between flower organ evolutionary modules of *Bulbophyllum* Clade *A* (38 spp.), for uncorrected and phylogenetically corrected datasets, respectively

	Correlation Coefficients (*ρ*s)	
Source of Correlation/Flower Organ Modules	Uncorrected	Phylogenetically Corrected
Within modules		
Sepals	0.28	0.28
Lateral petals	0.36	0.32
Labellum^1^	0.43	0.43
Column‐part^2^	0.53	0.46
Between modules		
Sepals vs. lateral petals	0.17	0.15
Sepals vs. labellum^1^	0.26	0.27
Sepals vs. column‐part^2^	0.16	0.14
Lateral petals vs. column‐part^2^	0.17	0.15
Lateral petals vs. labellum^1^	0.12	0.20
Labellum^1^ vs. column‐part^2^	0.12	0.15

*Note*: Estimates were obtained by emmli under the best‐fit developmental (“evo‐devo”) hypothesis H6*, with labellum and column‐foot treated together (see also Tables 2 and S3; Figs. 3 and S2).

^1^Including column‐foot

^2^Excluding‐column foot.

### MODES AND RATES OF MODULE EVOLUTION

Based on the preferred four‐module configuration of H6*, the PL analyses in rpanda provided unambiguous support for an OU model best‐fitting the 3D LM coordinates of three flower organ evolutionary modules, that is, sepals, lateral petals, and labellum + column‐foot, respectively (lowest GIC value in 100%, 71%, and 100% of the phylogenetic trees, respectively; Table 4). The mean estimates of *α* (± 2SD) recovered by the OU model for sepals (1.33 ± 0.7) and labellum + column‐foot (1.66 ± 0.7) were relatively small by common standards (<2; Beaulieu et al. 2012), whereas for the lateral petals this value was higher but also more variable (3.33 ± 4.8; Table 4). Nonetheless, the corresponding mean estimates of *t*_1/2_, ranging from 2.39 to 5.99 Myr (Table 4), were all much smaller than the tree height of Clade *A* (about 11.5 Myr). Hence, although being relatively weak, the three OU processes inferred are well distinguishable from the BM model (Beaulieu et al. 2012; Cooper et al. 2016). By contrast, for the column‐part, the BM model provided the best fit, receiving support in 80% of the trees tested (Table 4).

**Table 4:** Evolutionary model fitting to each of the four flower organ modules of *Bulbophyllum* Clade *A* (38 spp.), using a penalized likelihood (PL) approach (Clavel et al. 2019), as implemented in rpanda

Modules	Model	Parameter (Mean ± 2SD)	Phylogenetic Half‐Life (*t*_1/2_) in Myr	GIC (Mean ± 2SD)	ΔGIC (2.5%–97.5% Range)	Trees Preferred (%)
Sepals						
	BM	–	–	–11,263.06 ± 31	6.95–54.06	0
	EB	*r* = 1.75 × 10^–3^ ± 0.03	–	–11,261.08 ± 31	8.94–56.06	0
	**OU**	***α* = 1.33 ± 0.7**	**5.99**	**–11,288.03 ± 33**	**0**	**100**
Lateral petals						
	BM	–	–	–7112.27 ± 12	0–15.59	27
	EB	*r* = 0.16 ± 1.2	–	–7110.34 ± 12	0–17.59	2
	**OU**	***α* = 3.33 ± 4.8**	**2.39**	**–7116.53 ± 15**	**0–3.04**	**71**
Labellum^1^						
	BM	–	–	–7077.12 ± 19	1.06–48.44	0
	EB	*r* = 0	–	–7075.12 ± 19	3.06–50.44	0
	**OU**	***α* = 1.66 ± 0.7**	**4.80**	**–7098.02 ± 23**	**0**	**100**
Column‐part^2^						
	**BM**	–	–	**–9732.18 ± 17**	**0–6.38**	**80**
	EB	*r* = **–**1.73 × 10^–3^ ± 0.03	–	–9730.19 ± 17	1.23–8.37	0
	OU	*α* = 0.66 ± 0.6	–	–9730.84 ± 17	0–3.97	20

*Note*: Support for each model (i.e., BM, Brownian motion; EB, early burst; OU, single‐optimum Ornstein–Uhlenbeck) was evaluated over 100 trees. The latter were first randomly sampled from the Bayesian posterior distribution of the entire Malagasy *Bulbophyllum* phylogeny (Gamisch et al. 2021) and then pruned to the 38 species included in the present study. The four evolutionary modules were identified by emmli under the best‐fit modularity hypothesis H6*, with labellum and column‐foot treated together (see Tables 2 and S3; Figs. 3 and S2). For each module, namely, flower organ, the best‐fit evolutionary trait model, as determined by the lowest GIG value and the highest percentage of trees preferring this model, is highlighted in bold. For each of the three modules that were best fitting an OU model, the phylogenetic half‐life (*t*_1/2_) is reported (in million years, Myr). All models (BM, EB, OU) estimate **R**, the multivariate counterpart of *σ* ^2^⁠, and the diffusion parameter of the univariate BM model, whereas the EB and OU models estimate an extra parameter (*r* and *α*, respectively). Following Clavel et al. (2019), only the latter are shown here, whereas the Brownian parameters are given in the high‐dimensional **R** matrix (available upon request, for the whole dataset and each module separately, along with correlational heatmaps).

*r* = EB parameter of exponential rate decrease; *α* = rate of adaptation parameter for OU; GIC = Generalized Information Criterion; ratedifference in GIG value between the best model and the model being compared; SD = standard deviation.

^1^Including column‐foot

^2^Excluding‐column foot.

Using geomorph, we found that the column‐part had evolved at a significantly different (i.e., lower) net rate (*σ^2^*_mult_ = 1.192 × 10^–4^, *P* ≤ 0.027) when compared to the other three evolutionary modules. Among those, the sepals appeared to have the highest rate (4.368 × 10^–4^), followed by the labellum + column‐foot (3.741 × 10^–4^) and the lateral petals (2.024 × 10^–4^); however, none of these differences were statistically significant (pairwise *P* values ≥ 0.182), suggesting overall similar rates. We emphasize that these results obtained by geomorph should be interpreted with some caution as they rest on the program's default assumption of a BM process (which unlikely holds for the entire dataset; see above). Nonetheless, among the four modules, the column‐part also displayed the lowest value of disparity (in terms of Procrustes variance: 0.09 × 10^–2^), followed by the lateral petals (0.18 × 10^–2^), the labellum + column‐foot (0.21 × 10^–2^), and the sepals (0.39 × 10^–2^). However, based on pairwise comparisons among the four modules, only the sepals and the column‐part differed significantly in disparity (*P* = 0.033), whereas all other comparisons proved nonsignificant (all *P* values ≥ 0.28).

## Discussion

Evolutionary modularity of flower shape in species of Malagasy *Bulbophyllum* Clade *A* is best explained by developmental‐genetic factors (hypotheses H6 and especially H6*). This finding seems to contrast with most other studies of flower modularity, both at the intra‐ and interspecific levels, supporting instead functional‐adaptive hypotheses of attraction (e.g., Tucić et al. 2013) and especially efficiency (e.g., Carvallo and Medel 2005; Pérez et al. 2007; Bissell and Diggle 2008, 2010; Rosas‐Guerrero et al. 2011; Heywood et al. 2017; Armbruster and Wege 2019; Dellinger et al. 2019; Reich et al. 2020). In fact, these latter studies would suggest that functional adaptation to pollinators could be more important than developmental processes in shaping trait covariation within flowers (Esteve‐Altava 2017), especially in highly specialized pollination systems (e.g., Stebbins 1970; Cresswell 1998; Reich et al. 2020; but see Herrera et al. 2002; Armbruster and Wege 2019). However, the majority of these studies refer to variational (i.e., intraspecific) modularity; hence, whether the same trait correlations are replicated at the macroevolutionary level remains to be investigated. Nonetheless, we also found strong support for a “labellum + column‐foot” evolutionary module (H6*), which is better explained by function (see below), suggesting that not only developmental‐genetic factors but also functional adaptation could have played a role by shaping evolutionary trait covariation in Malagasy *Bulbophyllum*. Furthermore, as suggested by the evolutionary modular flower structure identified here, different suites of floral traits appear to have evolved independently from each other in these tropical orchids, which in turn should have promoted their floral morphological diversity (e.g., Goswami and Polly 2010; Diggle 2014; Larouche et al. 2018; Edie et al. 2022). Moreover, as discussed below, it seems likely that three of the four evolutionary modules identified (all except the column‐part) have been shaped by pollinator‐mediated selection.

### EVIDENCE FOR A FOUR‐MODULE (“EVO‐DEVO”) FLOWER CONFIGURATION IN *Bulbophyllum* CLADE *A*

The present study provides support for a four‐module flower configuration in *Bulbophyllum* Clade *A* at the macroevolutionary level. This particular evolutionary module structure is clearly evidenced by the strong support found for hypothesis H6 (treating sepals, lateral petals, labellum, column as separate modules), and even more so for its counterpart, (sub‐)hypothesis H6*, considering the column‐foot as part of the labellum module (Tables 1, 2, and S3; Figs. 3 and S2). This finding is remarkably in line with ample evidence from developmental‐genetic (‘evo‐devo’) studies on the orchid flower, indicating that the identity of the three sepals, the two lateral petals, the labellum, and the column is controlled by the differential expression of four classes of *DEF*‐like MADS‐box genes, together with other floral homoeotic genes (Mondragón‐Palomino and Theißen 2008, 2009, 2011; Mondragón‐Palomino 2013; Bateman and Rudall 2019; reviewed in Wang et al. 2019). Traditionally, the labellum has been viewed as a highly modified median petal (e.g., Arditti and Harrison 1979). In fact, the floral primordial initiation of the labellum usually follows immediately after the development of the lateral petals (e.g., in *Phalaenopsis*: Pramanik et al. 2020). Hence, in terms of floral ontogeny, the labellum clearly belongs to the inner perianth whorl (or “petal whorl”) but is nonetheless under specific genetic control (see above), which allowed this organ to evolve into very different and specialized morphologies (Mondragón‐Palomino and Theißen 2009; Gravendeel and Dirks‐Mulder 2015; Bateman and Rudall 2019). Moreover, together with the column, the different organ classes of the perianth are generally considered to represent developmentally conserved and constrained elements of the orchid flower (Mondragón‐Palomino and Theißen 2009; Madan et al. 2015; Bateman and Rudall 2019). However, in Malagasy *Bulbophyllum* Clade *A*, these constraints apparently did not prevent the joint evolution of two floral parts, which are generally thought to be under separate developmental/genetic control, namely, the labellum and the column‐foot (see further below).

### EVIDENCE FOR PREDOMINANTLY CONSTRAINED TRAIT EVOLUTION AT BOTH THE WITHIN‐ AND WHOLE‐FLOWER LEVELS

Based on our high‐dimensional PL‐based model testing in rpanda, an Ornstein–Uhlenbeck (OU) model of constrained evolution provided by far the best fit for the 3D LM data of three of the four evolutionary modules (i.e., sepals, lateral petals, and labellum + column‐foot), whereas the column‐part best‐fitted a neutral (BM) model (Table 4). Notably, in a previous study by Artuso et al. (2021), the OU model was found to best explain whole‐flower shape variation among *Bulbophyllum* Clade *A* species and interpreted to reflect pollinator‐driven (directional and/or stabilizing) selection (see also Serrano‐Serrano et al. 2015; Joly et al. 2018; Ibañez et al. 2019; Reich et al. 2020). In consequence, based on the present results, the same selective pressures might be invoked for sepals, lateral petals, and labellum + column‐foot. However, as for any OU‐driven phenotypic evolution, intrinsic (e.g., developmental, genetic) factors remain viable alternative explanations (e.g., Hansen 2014; see also Futuyma 2010; Blomberg et al. 2020). Nevertheless, there is evidence that the whole‐flower shapes of some Clade *A* species evolved independently toward different phenotypic (OU) optima (Artuso et al. 2021), suggesting that developmental/genetic factors are not the primary cause of constrained flower shape evolution in these orchids (see also Davis et al. 2014). Unfortunately, testing multi‐peak OU optima for high‐dimensional morphometric data, such as our 3D‐module dataset, is not yet achievable. However, when combined with the highly specialized “see‐saw” pollination mechanism of *Bulbophyllum* (see in the introductory text and Fig. 1), it seems plausible to hypothesize that OU‐driven evolution at both the whole‐ and within‐flower levels in Clade *A* (Artuso et al. 2021; this study) is most likely the result of selection exerted by the group's main pollinators (small Dipteran flies). On the other hand, the fact that the column‐part better fits a BM than an OU model reveals that this organ evolved in a neutral fashion (despite its relatively high integration and slow evolution, see below). This also concurs with several studies in animals, showing that morphological modular (sub)units can follow different modes of evolution, not only between each other, but also with respect to the higher level (“whole”) structure (e.g., Gómez‐Robles and Polly 2012; Hopkins and Lidgard 2012; Goswami and Finarelli 2016; Felice and Goswami 2018). Notably, a recent study of Asian *Bulbophyllum* (Hu et al. 2020) also supported different models of trait evolution for the two lateral sepals (BM) and the labellum (OU), suggesting that especially the latter organ is subject to different pollinator‐mediated selective pressures. However, this latter study focused on these two organs only, using a small number of continuous characters (linear measurements), which might explain why a different mode of evolution was found for the sepals (BM) as compared to the present, high‐dimensional modularity analysis (OU).

### EVIDENCE FOR INDEPENDENT EVOLUTION OF THE FOUR FLOWER MODULES IDENTIFIED ACROSS CLADE A

There are at least three lines of evidence suggesting that all four flower evolutionary modules identified herein evolved with some degree of independence across the Clade *A* phylogeny. First, in terms of correlation (“integration”) coefficients (*ρ* values), column and labellum + column‐foot were found to be the most integrated modules, and sepals and lateral petals the least. Nonetheless, *ρ* values were consistently lower between than within modules, testifying to their relative autonomy (Table 3). Second, when compared to the other three modules, the column‐part was found to have evolved at a significantly lower net rate (*σ* ^2^_mult_ = 1.192 × 10^–4^ vs. 2.024 × 10^–4^ to 4.368 × 10^–4^; *P* ≤ 0.027); likely as a consequence, this organ also displayed lower levels of morphological variation (disparity), especially in relation to the sepals (Procrustes variance: 0.09 × 10^–2^ vs. 0.39 × 10^–2^; *P* = 0.033). And third, based on the “rate of adaptation” (*α*) values of the OU model (Table 4), we may indirectly infer that both sepals and labellum + column‐foot were only slowly attracted (or “pulled”) toward their respective adaptive optima, whereas the lateral petals were more strongly attracted (Beaulieu et al. 2012; Voje and Hansen 2013). Together, these results provide evidence that all four evolutionary modules evolved largely independently, although most of them underly the same macroevolutionary (OU) process (except for the column‐part, BM; see above). This supports the idea that flower modularity in Malagasy *Bulbophyllum* could have enhanced the group's floral diversity, namely, “evolvability” (sensu Armbruster et al. 2014), by allowing each module to follow its own evolutionary trajectory, as likewise predicted by “evo‐devo” models of the orchid flower (e.g., Mondragón‐Palomino and Theißen 2008, 2009, 2011; Mondragón‐Palomino 2013; Bateman and Rudall 2019). Indeed, numerous studies corroborate the idea that evolutionary modularity is often associated with “higher evolvability,” namely, higher rates of trait evolution and disparity (Goswami and Polly 2010; Diggle 2014; Dellinger et al. 2019; Reich et al. 2020; see also Bardua et al. 2020 and references therein). However, the extent to which the modules identified herein are currently facilitating adaptive evolution in these tropical orchids, that is, “coordinated variation for selection to act upon” (cf. Armbruster et al. 2014), needs to be further investigated at the intraspecific level.

Strong developmental and/or genetic correlations are usually expected to slow down morphological trait evolution (Goswami and Polly 2010; Felice et al. 2018). In fact, several studies have found that highly integrated modules display both lower rates of evolution and reduced disparity (e.g., Claverie and Patek 2013; Felice and Goswami 2018; Martín‐Serra et al. 2019). Perhaps not unexpectedly, therefore, our results show that the column‐part, as the most integrated module (Table 3), also evolved more slowly and was less variable than the other evolutionary modules, especially those involved in pollinator attraction (i.e., sepals and lateral petals; see above). This might simply reflect the profound synorganization of male and female reproductive structures in this “super‐organ” (sensu Bateman and Rudall 2019) through congenital fusion, which could imply a high level of developmental‐genetic constraints per se (see also Rudall and Bateman 2002; Rudall et al. 2013). Of course, this does not mean that such integrated morphological structures cannot also show selective responses or even evolve in a neutral fashion (e.g., Goswami and Polly 2010; Conner 2012; Felice and Goswami 2018; Felice et al. 2018). In fact, based on the present results, the latter possibility might even be more likely for the column, given its best fit to a neutral BM model (Table 4). Considering the other flower traits, those involved in the mechanical fit to pollinators (here: the labellum) are generally expected to be under stronger selection and thus to be more integrated than those involved in attraction, such as the sepals and or petals (e.g., Ordano et al. 2008; Rosas‐Guerrero et al. 2011; Diggle 2014; Armbruster and Wege 2019; Reich et al. 2020). Accordingly, we found that the labellum was more integrated than the sepals and the lateral petals (Table 3), even though there were no significant differences among these three organ classes in terms of evolutionary rate and levels of disparity (all pairwise *P* values ≥ 0.18). Note, however, that the lateral sepals in *Bulbophyllum* might sometimes also serve as a landing platform (Hu et al. 2020). Overall, however, we have to acknowledge that the levels of within‐module integration reported herein are quite low (*ρ* ≤ 0.53; Table 3), implying that all modules could potentially evolve at a high rate and therefore achieve high disparity (Felice and Goswami 2018).

### IDENTIFICATION OF A SPECIFIC EVOLUTIONARY FLOWER MODULE IN *Bulbophyllum* ORCHIDS

Based on the best‐fit hypothesis H6*, we found strong evidence that the column‐foot covaried more during evolution with the labellum than with the rest of the column (i.e., column‐part), resulting in a specific “labellum + column‐foot” evolutionary module. We hypothesize that this covariation likely evolved due to functional selective constraints related to the peculiar “see‐saw” pollination mechanism of these orchids (see in the introductory text and Fig. 1). Along this line, selective forces exerted by the group's main pollinators (flies) must have been sufficiently strong to overcome developmental‐genetic constraints of both the labellum and the column by favoring a precise articulation, namely, morphological fit between the movable labellum and the column‐foot (e.g., Teixeira et al. 2004; Tan and Nishida 2005; Ong and Tam 2019; Hu et al. 2020). Notably, this “labellum + column‐foot” module displays correlation (“integration”) coefficients (*ρ* values) almost as high as those of the column‐part (Table 3). In addition, this module shows even higher evolutionary rate and disparity than the less integrated (attractive) lateral petals (see *Results*). The latter could suggest that pollinator‐mediated selection facilitated simultaneous adaptive changes in both the labellum (e.g., width, length) and the column‐foot (e.g., length, curvature), and thus likely improved the functionality of these floral structures without disrupting their finely coordinated articulation. Overall, the identification of this rather taxon‐specific evolutionary module lends support to the idea that functional trait integration can create new directions of change that align with the direction of selection, thereby facilitating morphological evolution (Conner 2012; Smith 2016; Bardua et al. 2020, 2019; Watanabe et al. 2019).

## Conclusion

Our main finding of a four‐module configuration underlying 3D flower shape evolution in *Bulbophyllum* Clade *A* lends support to morphogenetic (evo‐devo) studies, showing that the development of the orchid labellum is under distinct genetic control, with the potential to evolve (semi‐)independently from the lateral petals (e.g., Mondragón‐Palomino and Theißen 2009). Our results support current views that modular units within higher level structures in general, and within flowers in particular, can evolve independently of each other, which in turn should foster morphological diversity (disparity) and increase rates of phenotypic evolution (e.g., Bardua et al. 2020, 2019; Dellinger et al. 2019). Furthermore, our identification of a specific “labellum + column‐foot” evolutionary module agrees with the hypothesis that selection (here: probably pollinator‐mediated) can favor the integration of traits for functional purposes (Felice et al. 2018; Bardua et al. 2020, 2019; Watanabe et al. 2019; Martín‐Serra et al. 2021). Clearly, it would be interesting to uncover the modular organization of flowers in other orchid clades, and to verify whether the “labellum + column‐foot” module is also present in other orchid taxa with a movable labellum, namely, a “see‐saw” pollination mechanism (e.g., Pleurothallidinae: Karremans and Díaz‐Morales 2019; *Caladenia* spp.: Stoutamire 1983; Pridgeon et al. 2001). Ultimately, it might be possible to uncover which genes control interspecific variation in this particular module, and whether corresponding genotype‐phenotype maps across taxa reflect this “functional‐adaptive” modular unit (Melo et al. 2016; Porto et al. 2016).

## AUTHOR CONTRIBUTIONS

SA, AG, and HPC designed research. SA, AG, and YS performed research. SA and AG analyzed the data. AS and HPC wrote the manuscript. JS provided critical feedback and helped interpreting the results. All authors contributed to revisions and gave final approval for publication.

## CONFLICT OF INTEREST

The authors declare no conflict of interest.

## DATA ARCHIVING

Three‐dimensional image stacks of the raw flower scan data are deposited in TIFF format on the public repository of the University of Vienna (https://phaidra.univie.ac.at/o:1212007). All morphometric analyses were performed in R. Scripts are deposited in a .zip file on the public repository of the University of Vienna (https://phaidra.univie.ac.at/o:1594465).

Associate Editor: K.L. Voje

Handling Editor: M.L. Zelditch

## Supporting information

Supplementary information
