# Supplementary material for: Evidence for an evo‐devo‐derived hypothesis on three‐dimensional flower shape modularity in a tropical orchid clade
Source: Evolution. 2022 Oct 1;76(11):2587–604. doi: 10.1111/evo.14621 (PMC9828045; doi:10.1111/evo.14621)
Supplement: Supplementary file 1 — Supplementary information [file EVO-76-2587-s001.docx]

**Evidence for an evo-devo-derived hypothesis on 3D flower shape modularity in a tropical orchid clade**

**SUPPLEMENTARY MATERIAL**

**
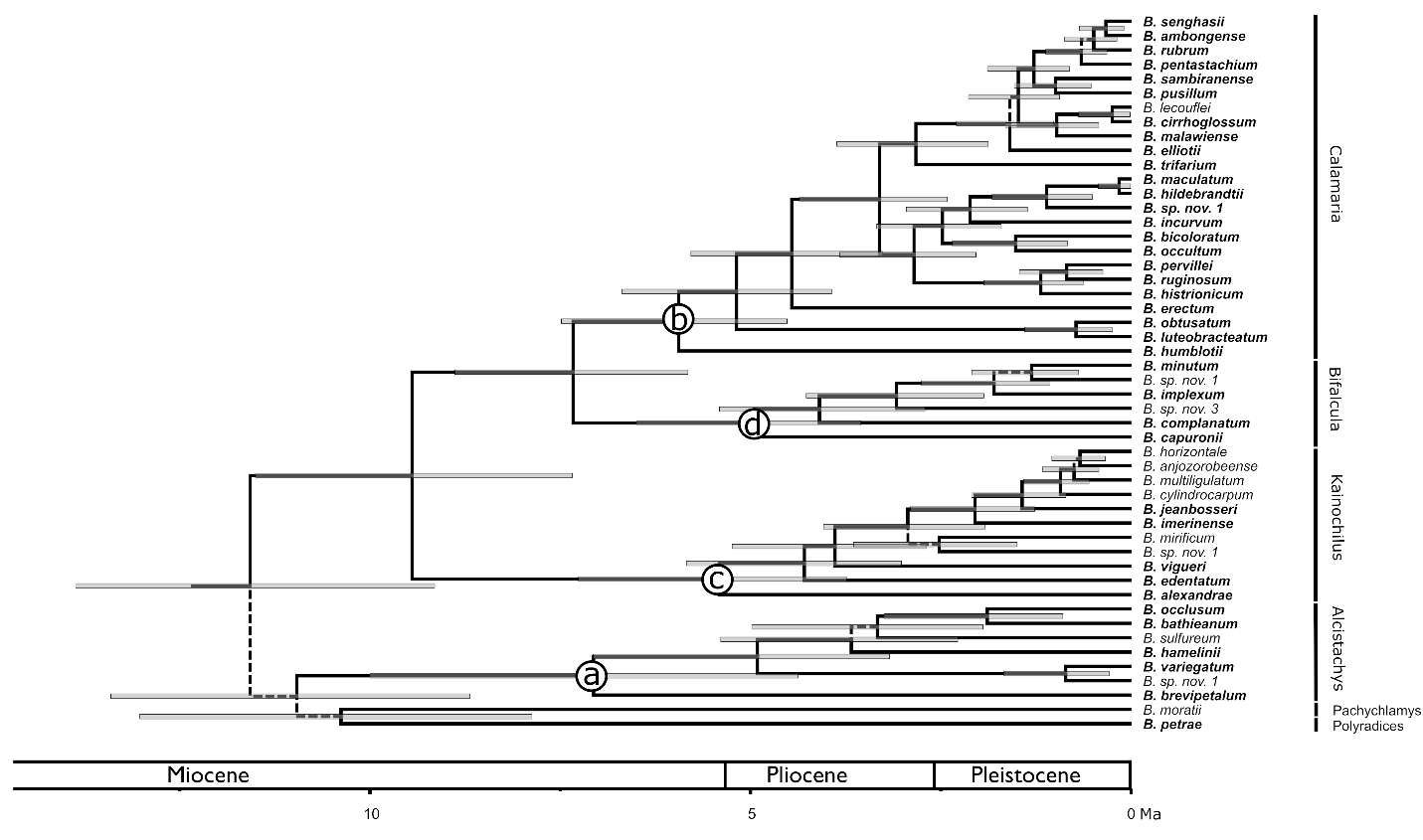
**

**Figure S1.** Maximum clade credibility (MCC) chronogram of Malagasy *Bulbophyllum* Clade *A* (50 spp.), based on concatenated DNA sequence data of three nuclear (*nr*ITS, *PI*, *Xdh*) and five plastid gene regions (*atp*I–*atp*H, *ycf1*, *mat*K, *trn*D–*trn*E, *psb*A–*trn*H; Gamisch et al. 2021). Branches with posterior probability (PP) ≥ 0.85 are marked as solid lines and those with PP < 0.85 as dotted lines. Grey bars indicate 95% highest posterior density (HPD) intervals around mean node ages (in million years ago, Ma). Sectional affiliations of species are shown on the right side (black bars). Letters a–d highlight the crown nodes of the four multi-species sections: (a) *Alcistachys*, (b) *Calamaria*, (c) *Kainochilus* and (d) *Bifalcula*. The 38 species included in this study are marked in bold.

**
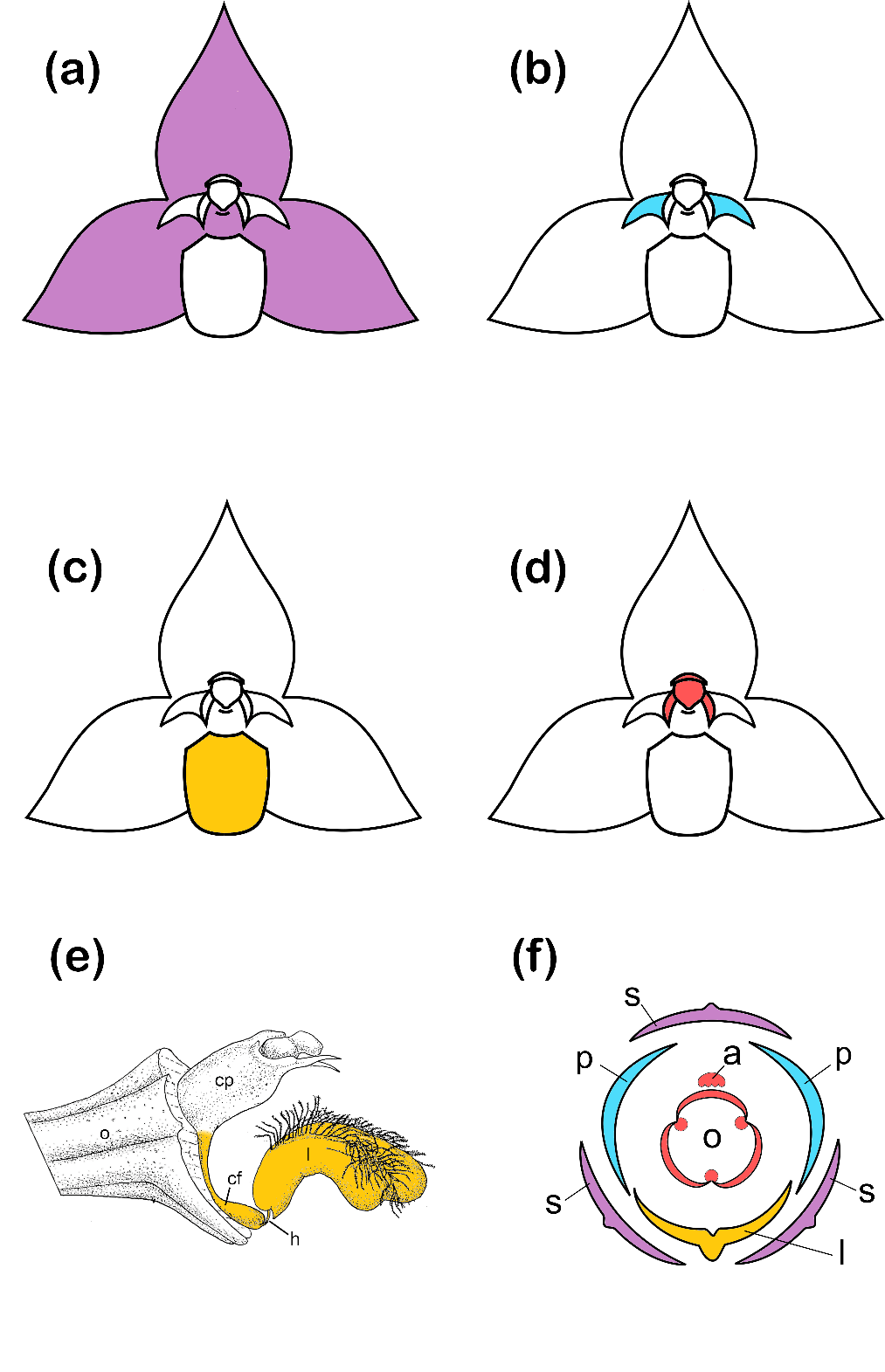
**

**Figure S2.** Illustration of the four flower evolutionary modules according to the best-fit developmental-genetic (‘evo-devo’) hypothesis H6* (with column-foot assigned to labellum), as identified by emmli (see **Tables 2** and **S3**). (a–d) Front view of resupinate flowers at anthesis, in horizontal orientation. Each flower sketch highlights in colour the flower organs belonging to each module: (a) sepals, (b) lateral petals, (c) labellum, and (d) column = gynostemium. (e) A flower in side view, with sepals and petals removed, highlighting (in yellow) the module of the labellum + column-foot (note that the ‘counterpart’ hypothesis H6 treats labellum and column as entirely separate units; see also **Fig. 3**). (f) Corresponding flower diagram with organs coloured accordingly to H6*. Abbreviations: a, anthers; cf, column-foot; cp, column-part (gynostemium); h, hinge; l, labellum (lip); o; ovary; p, petal; s, sepal. The sketch (e) was prepared by XX, based on a drawing of Malagasy *B. quadrifarium* by Juliet Beentje (Kew Gardens, UK).

**Table S1.** Sampling locations and voucher information for the 38 species of *Bulbophyllum* Clade *A* included in this study (see also Artuso et al. 2021). Further indicated are the two sample preparation methods used for high-resolution X-ray computed tomography (HRX-CT) scanning (IAA, in alcohol atmosphere; CPD, critical point drying), the number (and codes) of landmarks (LMs) estimated per species (where applicable).

| Section/species | | Sample  location ^1^ | Source (voucher) ^2^ | Collector ^3^ | Year of collection | Preparation method | Number (codes) of  LMs estimated ^4^ |
| --- | --- | --- | --- | --- | --- | --- | --- |
| ***Alcistachys*** Schltr. | |  |  |  |  |  |  |
|  | *B. bathieanum* Schltr. | M | X(FS 5124) | X | 2008 | IAA | – |
|  | *B. brevipetalum* H.Perrier | M | X(FS 2048) | X | 2005 | IAA | – |
|  | *B. hamelinii* W.Watson | M | X(FS 3257) | X | 2006 | IAA | 3 (11, 12, 13) |
|  | *B. occlusum* Ridl. | M | X(FS 1501) | X | 2004 | IAA | – |
|  | *B. variegatum* Thouars | RI | X(FS 799) | X | 2002 | IAA | 3 (11, 12, 13) |
| ***Bifalcula*** Schltr. | |  |  |  |  |  |  |
|  | *B. capuronii* Bosser | M | X(FS 1010) | X | 2002 | CPD | – |
|  | *B. complanatum* H.Perrier | M | X(FS 5756) | X | 2009 | IAA | – |
|  | *B. implexum* Jum. & H.Perrier | M | X(FS 6042) | X | 2009 | IAA | 1 (25) |
|  | *B. minutum* Thouars | M | X(FS 5306) | X | 2008 | CPD | – |
| ***Calamaria*** Schltr. | |  |  |  |  |  |  |
|  | *B. ambongense* Schltr. | M | X(FS 6022) | X | 2009 | IAA | – |
|  | *B. bicoloratum* Schltr. | M | X(FS 5841) | X | 2009 | IAA | 1 (13) |
|  | *B. cirrhoglossum* H.Perrier | M | X(FS 4125) | X | 2007 | CPD | – |
|  | *B. elliotii* Rolfe | M | X(FS 863) | X | 2002 | IAA | – |
|  | *B. erectum* Thouars | M | X(FS 5383) | X | 2009 | CPD | 3 (24, 11, 12) |
|  | *B. hildebrandtii* Rchb. f. | M | X(FS 5682) | X | 2009 | CPD | 1 (11) |
|  | *B. histrionicum* Rchb. f. ex G.A.Fisch. & P.J.Cribb | M | X(OR 1263_09) | X | 2009 | IAA | – |
|  | *B. humblotii* Rolfe ex Scott-Elliot | M | X(FS 1008) | X | 2002 | IAA | 12 (3, 20-23, 26–29, 32, 39, 41) |
|  | *B. incurvum* Thouars | RI | X(FS 1081) | X | 2002 | CPD | – |
|  | *B. luteobracteatum* Jum. & H.Perrier | M | X(FS 5501) | X | 2009 | IAA | – |
|  | *B. maculatum* Jum. & H.Perrier | M | X(FS 1133) | X | 2004 | IAA | 9 (14, 15, 24, 26, 27, 47, 49, 52, 53) |
|  | *B. malawiense* B.Morris | MAL | X(OR 146_05) | X | 2001 | IAA | – |
|  | *B. obtusatum* Schltr. | M | X(FS 5736) | X | 2009 | CPD | 3 (11, 14, 15) |
|  | *B. occultum* Thouars | M | X(FS 5028) | X | 2008 | CPD | – |
|  | *B. pervillei* Rolfe | M | X(FS 818) | X | 2002 | CPD | 2 (12, 13) |
|  | *B. pusillum* Thouars | M | X(FS 2019) | X | 2005 | CPD | 3 (24, 26, 27) |
|  | *B. pentastachium* (Pfitzer) Schltr. | M | X(FS 826) | X | 2002 | CPD | 1 (12) |
|  | *B. rubrum* Jum. & H.Perrier | M | X(M3522007) | X | Unknown | IAA | 4 (12, 3, 30, 42) |
|  | *B. ruginosum* H.Perrier | M | X(OR 1519_09) | X | 2009 | IAA | 1 (24) |
|  | *B. sambiranense* H.Perrier ex Hermans | M | X(FS 5718) | X | 2009 | CPD | – |
|  | *B.senghasii* G.A.Fisch. & Sieder | M | X(FS 3969) | X | 2006 | CPD | – |
|  | *B. sp. nov. 1* | M | X(OR 1271_09) | X | 2009 | IAA | – |
|  | *B. trifarium* Rolfe | M | X(OR 1576_09) | X | Unknown | CPD | – |
| ***Kainochilus*** Schltr. | |  |  |  |  |  |  |
|  | *B. alexandrae* Schltr. | M | X(FS 2205) | X | 2005 | IAA | – |
|  | *B. edentatum* H.Perrier | M | X(FS 866) | X | 2002 | IAA | – |
|  | *B. imerinense* Schltr. | M | X(FS 2991) | X | 2006 | IAA | – |
|  | *B. jeanbosseri* Gamisch & Hermans | M | X(FS 7342) | X | 2018 | IAA | 1 (3) |
|  | *B. viguieri* Schltr. | M | X(FS 2580) | X | 2005 | IAA | 3 (11, 12, 13) |
| ***Polyradices*** G.A.Fisch., Sieder & P.J.Gribb | | | | | | | |
|  | *B. petrae* G.A.Fisch., Sieder &P.J.Gribb | M | X(FS 2287) | X | 2000 | IAA | – |

^1^ Locality codes: M, Madagascar; MAL, Malawi, RI, Réunion Island (France).

^2^ Source: X: Anonymized for peer reviewing.

^3^ Collector abbreviations: X: Anonymized for peer reviewing.

^4^ See Artuso et al. (2021) for details on how missing LMs were estimated, and **Table S2** for identification of LM codes; “–“, all 52 LMs could be placed without adjustment.

**Table S2.** Description of the 52 landmarks (LMs 1–52), including 38 discrete and 14 semi-LMs, and their assignment to each flower evolutionary module according to the ten evolutionary modularity hypotheses (‘H’) tested across *Bulbophyllum* Clade *A* (38 ssp.). Note, these hypotheses include six major ones (H1–6) as well as four ‘counterparts’ (H2*, H4*, H5* and H6*) that treat labellum and column-foot together (see text, **Tables 2** and **S3**; **Figs 3** and **S2**). Codes M1–M4 indicate the assignment of a given LM to the different modules (‘M’) specified per hypothesis, as identified in Tables 2 and S3 (e.g. M1 = LM assigned to module 1; M2 = LM assigned to module 2, etc.). See Artuso et al. (2021) for details on landmark definition and placement, and **Figure 2** for an exemplary illustration of landmark positions on a flower scan of Malagasy *B. francoisii.* Note, the terms adaxial and abaxial are used here to indicate the position of the LMs in the organs with respect to the inflorescence axis of the flower, at the resupinate stage.

| LM code | Description | H1 | H2 | H2* | H3 | H4 | H4* | H5 | H5* | H6 | H6* |
| --- | --- | --- | --- | --- | --- | --- | --- | --- | --- | --- | --- |
| 1 | Adaxial side of the flower base | M1 | M1 | M1 | M1 | M1 | M1 | M1 | M1 | M1 | M1 |
| 2 | Abaxial side of the flower base | M2 | M3 | M3 | M2 | M2 | M2 | M3 | M3 | M4 | M4 |
| 3 | Starting point of the stylar canal in the middle section | M2 | M3 | M3 | M2 | M2 | M2 | M3 | M3 | M4 | M4 |
| 4 | Tip of the frontal conjunction between the right and left (lateral) sepals | M1 | M1 | M1 | M1 | M1 | M1 | M1 | M1 | M1 | M1 |
| 5 | Abaxial bulge of the right sepal base | M1 | M1 | M1 | M1 | M1 | M1 | M1 | M1 | M1 | M1 |
| 6 | Abaxial bulge of the left sepal base | M1 | M1 | M1 | M1 | M1 | M1 | M1 | M1 | M1 | M1 |
| 7 | Adaxial bulge of the right sepal base | M1 | M1 | M1 | M1 | M1 | M1 | M1 | M1 | M1 | M1 |
| 8 | Adaxial bulge of the left sepal base | M1 | M1 | M1 | M1 | M1 | M1 | M1 | M1 | M1 | M1 |
| 9 | Right bulge of the dorsal sepal base | M1 | M1 | M1 | M1 | M1 | M1 | M1 | M1 | M1 | M1 |
| 10 | Left bulge of the dorsal sepal base | M1 | M1 | M1 | M1 | M1 | M1 | M1 | M1 | M1 | M1 |
| 11 | Tip of the right sepal | M1 | M1 | M1 | M1 | M1 | M1 | M1 | M1 | M1 | M1 |
| 12 | Tip of the left sepal | M1 | M1 | M1 | M1 | M1 | M1 | M1 | M1 | M1 | M1 |
| 13 | Tip of the dorsal sepal | M1 | M1 | M1 | M1 | M1 | M1 | M1 | M1 | M1 | M1 |
| 14 | Semi-LM between LMs 7 and 11 | M1 | M1 | M1 | M1 | M1 | M1 | M1 | M1 | M1 | M1 |
| 15 | Semi-LM between LMs 5 and 11 | M1 | M1 | M1 | M1 | M1 | M1 | M1 | M1 | M1 | M1 |
| 16 | Semi-LM between LMs 8 and 12 | M1 | M1 | M1 | M1 | M1 | M1 | M1 | M1 | M1 | M1 |
| 17 | Semi-LM between LMs 6 and 12 | M1 | M1 | M1 | M1 | M1 | M1 | M1 | M1 | M1 | M1 |
| 18 | Semi-LM between LMs 9 and 13 | M1 | M1 | M1 | M1 | M1 | M1 | M1 | M1 | M1 | M1 |
| 19 | Semi-LM between LMs 10 and 13 | M1 | M1 | M1 | M1 | M1 | M1 | M1 | M1 | M1 | M1 |
| 20 | Adaxial side of the right petal base | M1 | M1 | M1 | M2 | M1 | M1 | M2 | M2 | M2 | M2 |
| 21 | Adaxial side of the left petal base | M1 | M1 | M1 | M2 | M1 | M1 | M2 | M2 | M2 | M2 |
| 22 | Abaxial side of the right petal base | M1 | M1 | M1 | M2 | M1 | M1 | M2 | M2 | M2 | M2 |
| 23 | Abaxial side of the left petal base | M1 | M1 | M1 | M2 | M1 | M1 | M2 | M2 | M2 | M2 |
| 24 | Tip of the right petal | M1 | M1 | M1 | M2 | M1 | M1 | M2 | M2 | M2 | M2 |
| 25 | Tip of the left petal | M1 | M1 | M1 | M2 | M1 | M1 | M2 | M2 | M2 | M2 |
| 26 | Semi-LM between LMs 20 and 24 | M1 | M1 | M1 | M2 | M1 | M1 | M2 | M2 | M2 | M2 |
| 27 | Semi-LM between LMs 22 and 24 | M1 | M1 | M1 | M2 | M1 | M1 | M2 | M2 | M2 | M2 |
| 28 | Semi-LM between LMs 21 and 25 | M1 | M1 | M1 | M2 | M1 | M1 | M2 | M2 | M2 | M2 |
| 29 | Semi-LM between LMs 23 and 25 | M1 | M1 | M1 | M2 | M1 | M1 | M2 | M2 | M2 | M2 |
| 30 | Base of the stigmatic cavity along the median section | M2 | M3 | M3 | M2 | M2 | M2 | M3 | M3 | M4 | M4 |
| 31 | Meeting point between the rostellum and the clinandrium | M2 | M3 | M3 | M2 | M2 | M2 | M3 | M3 | M4 | M4 |
| 32 | Distal point of the rostellum along the median section | M2 | M3 | M3 | M2 | M2 | M2 | M3 | M3 | M4 | M4 |
| 33 | Conjunction-point between the column and the dorsal sepal, along the median section | M1 | M1 | M1 | M1 | M1 | M1 | M1 | M1 | M1 | M1 |
| 34 | Tip of the hinge between the column and the anther cap | M2 | M3 | M3 | M2 | M2 | M2 | M3 | M3 | M4 | M4 |
| 35 | Right side of the hinge between the column and the anther cap | M2 | M3 | M3 | M2 | M2 | M2 | M3 | M3 | M4 | M4 |
| 36 | Left side of the hinge between the column and the anther cap | M2 | M3 | M3 | M2 | M2 | M2 | M3 | M3 | M4 | M4 |
| 37 | Base of the right stelidium | M2 | M3 | M3 | M2 | M2 | M2 | M3 | M3 | M4 | M4 |
| 38 | Base of the left stelidium | M2 | M3 | M3 | M2 | M2 | M2 | M3 | M3 | M4 | M4 |
| 39 | Tip of the right stelidium | M2 | M3 | M3 | M2 | M2 | M2 | M3 | M3 | M4 | M4 |
| 40 | Tip of the left stelidium | M2 | M3 | M3 | M2 | M2 | M2 | M3 | M3 | M4 | M4 |
| 41 | Lower rim of the stigmatic cavity | M2 | M3 | M3 | M2 | M2 | M2 | M3 | M3 | M4 | M4 |
| 42 | Point where ligament is attached to the column | M2 | M3 | M2 | M2 | M2 | M1 | M3 | M2 | M4 | M3 |
| 43 | Semi-LM between LMs 41 and 42 | M2 | M3 | M2 | M2 | M2 | M1 | M3 | M2 | M4 | M3 |
| 44 | Point of ligament attachment to the labellum | M2 | M2 | M2 | M2 | M1 | M1 | M2 | M2 | M3 | M3 |
| 45 | Point of maximal curvature of the labellum on its abaxial side, along the median section | M2 | M2 | M2 | M2 | M1 | M1 | M2 | M2 | M3 | M3 |
| 46 | Distal end of the labellum | M2 | M2 | M2 | M2 | M1 | M1 | M2 | M2 | M3 | M3 |
| 47 | Point of maximal curvature of the labellum on its adaxial side, along the median section | M2 | M2 | M2 | M2 | M1 | M1 | M2 | M2 | M3 | M3 |
| 48 | Semi-LM between LMs 46 and 47 | M2 | M2 | M2 | M2 | M1 | M1 | M2 | M2 | M3 | M3 |
| 49 | Point of maximal curvature of the labellum’s right lateral lobe | M2 | M2 | M2 | M2 | M1 | M1 | M2 | M2 | M3 | M3 |
| 50 | Point of maximal curvature of the labellum’s left lateral lobe | M2 | M2 | M2 | M2 | M1 | M1 | M2 | M2 | M3 | M3 |
| 51 | Semi-LM between LMs 46 and 49 | M2 | M2 | M2 | M2 | M1 | M1 | M2 | M2 | M3 | M3 |
| 52 | Semi-LM between LMs 46 and 50 | M2 | M2 | M2 | M2 | M1 | M1 | M2 | M2 | M3 | M3 |

**Table S3.** emmli-derived results for a total of ten flower evolutionary modularity hypotheses, plus a null model of ‘no modularity’, tested for the phylogenetically-corrected 3D-landmark dataset of *Bulbophyllum* Clade *A* (38 spp.). Each modularity hypothesis was tested for alternative parameterizations (a, b; or a–d), proposing that correlation (‘integration’) coefficients (ρ*s*) are the same or different within and/or between modules (Goswami and Finarelli, 2016). The best-fit hypothesis, as determined through the lowest corrected Akaike Information Criterion (AICc) score, is highlighted in bold, i.e. the developmental (‘evo-devo’) hypothesis H6*, with labellum and column-foot treated together, and hypothesizing different ρ values within and between modules (H6*­­­–d). For each hypothesis, module numbers (M1, M2, etc.) reflect the partitioning of landmarks, as detailed in **Table S2**. Corresponding results based on the uncorrected dataset can be found in **Table 2**.

| Hyp.  no. ^1^ | Category | Parameter  scheme ^2^ | Modular structure description | No. of  modules | *MaxL* | *K* | AICc | ΔAICc | Model PP |
| --- | --- | --- | --- | --- | --- | --- | --- | --- | --- |
| 0 | No modularity | – | – | – | 965.167 | 2 | -1926.33 | 478.415 | 1.30E-104 |
|  |  |  |  |  |  |  |  |  |  |
| 1 | Efficiency |  | M1 [sepals + lateral petals], M2 [labellum + column] | 2 |  |  |  |  |  |
|  |  | a |  |  | 995.206 | 3 | -1984.40 | 420.346 | 5.28E-92 |
|  |  | b |  |  | 1011.358 | 4 | -2014.69 | 390.055 | 2.00E-85 |
| 2 | Efficiency |  | M1 [sepals + lateral petals], M2 [labellum], M3 [column] | 3 |  |  |  |  |  |
|  |  | a |  |  | 1024.836 | 3 | -2043.65 | 361.088 | 3.90E-79 |
|  |  | b |  |  | 1119.265 | 5 | -2228.49 | 176.256 | 5.33E-39 |
|  |  | c |  |  | 1048.230 | 5 | -2086.41 | 318.327 | 7.52E-70 |
|  |  | d |  |  | 1142.660 | 7 | -2271.23 | 133.507 | 1.02E-29 |
| 2* | Efficiency |  | M1 [sepals + lateral petals], M2 [labellum (+ column-foot)], M3 [column-part] | 3 |  |  |  |  |  |
|  |  | a |  |  | 1032.957 | 3 | -2059.90 | 344.846 | 1.31E-75 |
|  |  | b |  |  | 1137.755 | 5 | -2265.46 | 139.277 | 5.71E-31 |
|  |  | c |  |  | 1068.969 | 5 | -2127.89 | 276.848 | 7.64E-61 |
|  |  | d |  |  | 1173.767 | 7 | -2333.45 | 71.291 | 3.31E-16 |
| 3 | Efficiency |  | M1 [sepals], M2 [lateral petals + labellum + column] | 2 |  |  |  |  |  |
|  |  | a |  |  | 992.743 | 3 | -1979.47 | 425.273 | 4.50E-93 |
|  |  | b |  |  | 995.675 | 4 | -1983.32 | 421.421 | 3.09E-92 |
| 4 | Attraction |  | M1 [sepals + lateral petals + labellum], M2 [column] | 2 |  |  |  |  |  |
|  |  | a |  |  | 1042.923 | 3 | -2079.83 | 324.914 | 2.79E-71 |
|  |  | b |  |  | 1082.653 | 4 | -2157.28 | 247.466 | 1.83E-54 |
| 4* | Attraction |  | M1 [sepals + lateral petals + labellum (+ column-foot)], M2 [column-part] | 2 |  |  |  |  |  |
|  |  | a |  |  | 1061.506 | 3 | -2116.99 | 287.748 | 3.28E-63 |
|  |  | b |  |  | 1129.049 | 4 | -2250.07 | 154.672 | 2.59E-34 |
| 5 | Development |  | M1 [sepals], M2 [petals, including labellum], M3 [column] | 3 |  |  |  |  |  |
|  |  | a |  |  | 1080.324 | 3 | -2154.63 | 250.111 | 4.89E-55 |
|  |  | b |  |  | 1098.464 | 5 | -2186.88 | 217.858 | 4.93E-48 |
|  |  | c |  |  | 1090.045 | 5 | -2170.04 | 234.697 | 1.09E-51 |
|  |  | d |  |  | 1108.185 | 7 | -2202.28 | 202.457 | 1.09E-44 |
| 5* | Development |  | M1 [sepals], M2 [petals, including labellum (+ column-foot)], M3 [column-part] | 3 |  |  |  |  |  |
|  |  | a |  |  | 1083.506 | 3 | -2161.00 | 243.746 | 1.18E-53 |
|  |  | b |  |  | 1126.682 | 5 | -2243.32 | 161.423 | 8.86E-36 |
|  |  | c |  |  | 1104.101 | 5 | -2198.16 | 206.584 | 1.38E-45 |
|  |  | d |  |  | 1147.277 | 7 | -2280.47 | 124.273 | 1.03E-27 |
| 6 | Development |  | M1 [sepals], M2 [lateral petals], M3 [labellum], M4 [column] | 4 |  |  |  |  |  |
|  |  | a |  |  | 1107.158 | 3 | -2208.30 | 196.444 | 2.20E-43 |
|  |  | b |  |  | 1150.536 | 6 | -2289.01 | 115.733 | 7.39E-26 |
|  |  | c |  |  | 1136.355 | 8 | -2256.60 | 148.140 | 6.79E-33 |
|  |  | d |  |  | 1179.733 | 11 | -2337.27 | 67.475 | 2.23E-15 |
| **6*** | **Development** |  | **M1 [sepals], M2 [lateral petals], M3 [labellum (+ column-foot)], M4 [column-part]** | **4** |  |  |  |  |  |
|  |  | a |  |  | 1122.838 | 3 | -2239.66 | 165.082 | 1.42E-36 |
|  |  | b |  |  | 1169.647 | 6 | -2327.23 | 77.512 | 1.47E-17 |
|  |  | c |  |  | 1166.663 | 8 | -2317.22 | 87.525 | 9.87E-20 |
|  |  | **d** |  |  | **1213.471** | **11** | **-2404.74** | **0** | **1.000** |

^1^ Hyp. no., modularity hypothesis number; those with an asterisk indicate hypotheses that assign landmarks (LMs) of the column-foot to the labellum.

^2^ Parameterization scheme of correlation (‘integration’) coefficients (ρs) within and between modules (with emmli connotations in italics): a, same within-module ρs and same between-module ρs (*same.Mod + same.between*); b, separate within-module ρs and same between-module ρs (*sep.Mod + same.between*); c, same within-module ρs and separate between-module ρs (*same.Mod + sep.between);* d, separate within-module ρs and separate between-module ρs (*sep.Mod + sep.between*).

*MaxL*, maximum log-likelihood; *K*, model parameters; AICc, finite sample corrected Akaike Information Criterion; ΔAICc, difference in AICc value between the best model and the model being compared; Model PP, model posterior probability (note, same as model log-likelihood; not shown).
